# Plasma matrix metalloproteinase-3 predicts mortality in acute respiratory distress syndrome: a biomarker analysis of a randomized controlled trial

**DOI:** 10.1186/s12931-023-02476-5

**Published:** 2023-06-22

**Authors:** Timothy W. Jones, Sultan Almuntashiri, Aaron Chase, Abdullah Alhumaid, Payaningal R. Somanath, Andrea Sikora, Duo Zhang

**Affiliations:** 1 Department of Pharmacy, Augusta University Medical Center, 1120 15th St., Augusta, GA 30912 USA; 2 Department of Clinical and Administrative Pharmacy, University of Georgia College of Pharmacy, 120 15th Street, HM-117, Augusta, GA 30912 USA

**Keywords:** Acute respiratory distress syndrome, Acute lung injury, Biomarker, Matrix metalloproteinase-3, Mortality prediction

## Abstract

**Background:**

Matrix metalloproteinase-3 (MMP-3) is a proteolytic enzyme involved in acute respiratory distress syndrome (ARDS) pathophysiology that may serve as a lung-specific biomarker in ARDS.

**Methods:**

This study was a secondary biomarker analysis of a subset of Albuterol for the Treatment of Acute Lung Injury (ALTA) trial patients to determine the prognostic value of MMP-3. Plasma sample MMP-3 was measured by enzyme-linked immunosorbent assay. The primary outcome was the area under the receiver operating characteristic (AUROC) of MMP-3 at day 3 for the prediction of 90-day mortality.

**Results:**

A total of 100 unique patient samples were evaluated and the AUROC analysis of day three MMP-3 showed an AUROC of 0.77 for the prediction of 90-day mortality (95% confidence interval: 0.67–0.87), corresponding to a sensitivity of 92% and specificity of 63% and an optimal cutoff value of 18.4 ng/mL. Patients in the high MMP-3 group (≥ 18.4 ng/mL) showed higher mortality compared to the non-elevated MMP-3 group (< 18.4 ng/mL) (47% vs. 4%, p < 0.001). A positive difference in day zero and day three MMP-3 concentration was predictive of mortality with an AUROC of 0.74 correlating to 73% sensitivity, 81% specificity, and an optimal cutoff value of + 9.5 ng/mL.

**Conclusions:**

Day three MMP-3 concentration and difference in day zero and three MMP-3 concentrations demonstrated acceptable AUROCs for predicting 90-day mortality with a cut-point of 18.4 ng/mL and + 9.5 ng/mL, respectively. These results suggest a prognostic role of MMP-3 in ARDS.

**Supplementary Information:**

The online version contains supplementary material available at 10.1186/s12931-023-02476-5.

## Introduction

Acute respiratory distress syndrome (ARDS) is a lethal disease without laboratory-guided diagnostic or prognostic biomarkers [1, 2]. The LUNG SAFE study determined clinicians failed to recognize ARDS 40% of the time, and only 34% recognized the disease at the first time fulfillment of ARDS diagnostic criteria [3]. This failure to recognize ARDS is problematic because early treatment has been associated with better response to ARDS therapies [4, 5]. Significant clinical heterogeneity exists among ARDS presentations, a factor likely contributing to this high rate of under recognition [6]. Given that delayed diagnosis of ARDS is common and may result in therapy initiation beyond the window for efficacy, rapid, objective tools for identifying the broad range of ARDS presentations are needed. Additionally, beyond diagnosis, failure to appropriately prognosticate the severity of illness may inhibit clinical-decision making regarding the use of invasive therapies most likely to benefit certain phenotypes (e.g., neuromuscular blockade, prone positioning).

Identification of ARDS sub-phenotypes using biomarkers has been proposed, but these efforts have primarily relied on non-specific biomarkers, such as inflammatory cytokines (e.g., IL-6, IL-1B, TNFa), which may represent general critical illness common to ARDS [7]. Recently, a lung-specific biomarker, club cell secretory protein (CC16), demonstrated reasonable AUROC for prediction of ARDS, as well as 60-day mortality in patients, from the FACTT trial [8]. This finding along with corroborating evidence, suggests phenotyping through combining lung-specific biomarkers, non-specific biomarkers, and physiological parameters may contribute substantially to bedside diagnostic and prognostic tools [9, 10]. The recent decades of ARDS research have sought to establish “biologically treatable traits” to simplify selecting patients likely to benefit from therapy, and single biomarkers, if capable of representing a combination of specific physiologic and biologic traits and readily available, will have clinical application [11].

Matrix metalloproteinases (MMPs) are extracellular proteases capable of degrading every part of the extracellular matrix and the proteins of the alveolar epithelial-endothelial unit under pro-inflammatory conditions, a process central to ARDS pathophysiology [12, 13]. Previous studies suggest serum and bronchoalveolar lavage fluid levels of MMP-3 may serve as a biomarker to inform targeted therapies in early ARDS [14–19]. Mice deficient in *Mmp-3* have less severe lung injury in acute lung injury (ALI) models [16, 20], and recently, early elevations in MMP-3 have been identified with COVID-19 observing the most prominent MMP-3 elevations in severe disease [21, 22].

Given the evidence supporting MMPs as contributors to ARDS pathophysiology, this study sought to explore the relationship of MMP-3 changes early in ARDS with patient outcomes in the context of a robust randomized controlled trial of ARDS patients, Albuterol to Treat Acute Lung Injury (ALTA). The study hypothesized that elevated MMP-3 from both static and dynamic measures would be associated with increased mortality.

## Materials and methods

This study was a secondary analysis of the multicenter randomized controlled trial, ALTA). ALTA included 282 mechanically ventilated patients and compared the beta-2-agonist albuterol to placebo for the treatment of acute lung injury (ALI)/ARDS [23]. This study was approved by the Augusta University Institutional Review Board (1128838-14).

Plasma MMP-3 concentrations were measured in 100 plasma samples from ALTA and 20 healthy control plasma samples using enzyme-linked immunosorbent assay (ELISA). The primary outcome was the area-under-the-receiver operating characteristic (AUROC) of day 3 MMP-3 concentrations to predict 90-day mortality in patients with ARDS. Day 0 and 3 were chosen because they approximated the baseline expression close to ARDS diagnosis and then reassessed several days into disease progression to allow discrimination between rapidly improving ARDS phenotypes described as rapidly improving by the 24-h mark [3, 24]. Secondary outcomes included the predictive value of the dynamic change (defined as the positive or negative absolute change) between day 0 (MMP-3 concentration at trial enrollment) and day 3 MMP-3 concentrations (MMP-3 concentration on the third day of trial enrollment) for 90-day mortality measured by AUROC and the association of MMP-3 concentration on APACHE III. Both day 3 and dynamic MMP-3 concentrations were evaluated for other patient outcomes, including ventilator-free days (VFDs) and ICU-free days. The diagnostic value of day 0 MMP-3 was also assessed via AUROC analysis using healthy patient control and ALTA ARDS plasma samples.

### Plasma samples

Plasma samples and coded data sheets from patients enrolled in ALTA were obtained from the National Heart, Lung, and Blood Institute’s (NHLBI) Biological Specimen and Data Repository Information Coordinating Center (BioLINCC). As negative controls, an additional 20 healthy patient plasma samples were obtained from Innovative Research Inc, Novi, MI. Samples were stored at − 80 °C. Plasma MMP-3 concentration was assessed in duplicates on days 0 and 3 by ELISA.

### Plasma total MMP-3 Protein Measurement using ELISA

All plasma samples were stored at − 80 °C until use. Plasma MMP-3 concentrations were measured with Human Total MMP-3 DuoSet ELISA Kit from R&D Systems, Inc, Catalog #: DY513 (Minneapolis, MN). Briefly, 100 μL of the sample (or control standard) and Reagent Diluent were added to each well. The plate was covered with an adhesive strip and incubated for 2 h at room temperature. Wells were aspirated and washed with Wash Buffer, repeating the wash process two times for a total of three washes. A 100 μL of the detection antibody in reagent diluent was added to each well. The plate was covered with a new adhesive strip and incubated for 2 h at room temperature. The aspiration and wash process was repeated three times. Then, 100 μL of the working dilution of Streptavidin-HRP was added to each well, and the plate was covered and incubated for 20 min at room temperature, followed by repeat aspiration and wash cycles. Following aspiration and wash, a 100 μL of substrate solution was added to each well and incubated for 20 min at room temperature. Lastly, add 50 μL of stop-solution (2N sulfuric acid) to each well. Optical density was determined at 450 nm. MMP-3 concentration was calculated based on a linear standard curve.

### Statistical analysis

Statistical analyses and figure development were performed with IBM SPSS Statistics Version 28.0. Statistical significance was assessed by a two-sided alpha of 0.05. Continuous variables were analyzed with Student’s t-test or Mann–Whitney U Test for parametric and non-parametric data, respectively. Categorical variables were assessed with Fischer’s Exact Test. A Shapiro–Wilk Test was performed to assess for normally distributed data with a significance of p < 0.05, indicating non-normal distribution. AUROC was calculated on ALTA samples dichotomized by the presence of 90-day mortality to assess the predictive capability of MMP-3 concentration for mortality. The optimal cutoff value for MMP-3 concentration was determined by calculating Youden’s index (YI). Logistic regression was performed in a backward stepwise fashion. The following variables were included in the original model: Apache III score, vasopressor use within the 24 h before randomization, PaO_2_/FiO_2_ at randomization, sex, body mass index, and day 3 MMP-3. At each step, the variable with the highest p-value was removed until all remaining variables had a p-value of 0.1 or less. Multicollinearity was excluded with variance inflation factors for each variable and goodness-of-fit was assessed with the Hosmer–Lemeshow test. Kaplan–Meier plots were used to estimate the survival rate in each group.

## Results

### Patient characteristics

The plasma concentration of MMP-3 was determined at day 0 and day 3 in 100 samples from ALTA (50 in the albuterol treatment group and 50 in the placebo group). Baseline characteristics did not differ between albuterol and treatment groups of the ALTA trial (Table 1). Most samples were derived from patients with pneumonia or sepsis as the ARDS etiology. ARDS severity was moderate in each group and comparable between placebo and albuterol groups (PaO_2_/FiO_2_ 140 vs. 144, p = 0.77.) The demographics and outcomes data based on the ALTA trial treatment group (albuterol vs. placebo) are included in the electronic supplement (see Additional file 2: Table S1).

**Table 1:** Demographics by MMP-3 level and change in MMP-3 from day 0 to 3

	Day 3 MMP-3 concentration			Day 0 to 3 MMP-3 difference		
	High (≥ 18.4 ng/mL) (n = 50)	Low (< 18.4 ng/mL) (n = 50)	P-value	High (≥ 9.4 ng/mL) (n = 33)	Low (< 9.4 ng/mL) (n = 67)	P-value
Characteristic						
Age (years)	55 ± 15	46 ± 15	0.003	57 ± 14	47 ± 15	0.001
Male	32 (64)	22 (44)	0.07	19 (58)	35 (52)	0.67
Body mass index	28 ± 6	28 ± 7	0.73	28 ± 6	28 ± 7	0.66
APACHE III, mean (SD)	106 ± 28	79 ± 23	0.001	107 ± 29	85 ± 26	< 0.001
Vasoactive use within 24 h before randomization	29 (58)	23 (46)	0.32	20 (61)	32 (47)	0.29
Time from ALI to randomization (hours), median	26 (13–37)	15 (10–28)	0.025	26.5 (13.2–38.4)	18.4 (10.2–28.7)	0.14
PaO_2_/FiO_2_	140 ± 63	144 ± 57	0.73	131 ± 60	148 ± 59	0.21
ARDS causes, n (%)						
Pneumonia	18 (36)	20 (40)	0.68	22 (44)	16 (32)	0.22
Sepsis	18 (36)	10 (20)	0.075	12 (24)	16 (32)	0.37
Aspiration	9 (18)	7 (14)	0.59	4 (8)	12 (24)	0.03
Trauma	4 (8)	6 (12)	0.5	8 (16)	2 (4)	0.046
Multiple transfusions	1 (2)	1 (2)	1.0	2 (4)	0	0.56
Other	0	6 (12)	0.047	2 (4)	4 (8)	0.4

All data are presented as n (%), mean ± SD, and median (interquartile range) unless otherwise noted

ALI: acute lung injury; ARDS: acute respiratory distress syndrome; APACHE III: Acute Physiology and Chronic Health Evaluation III; ICU: intensive care unit; MMP-3: matrix metalloproteinase-3; Vfd: ventilator free days

### MMP-3 as a prognostic marker

**Fig. 1:**
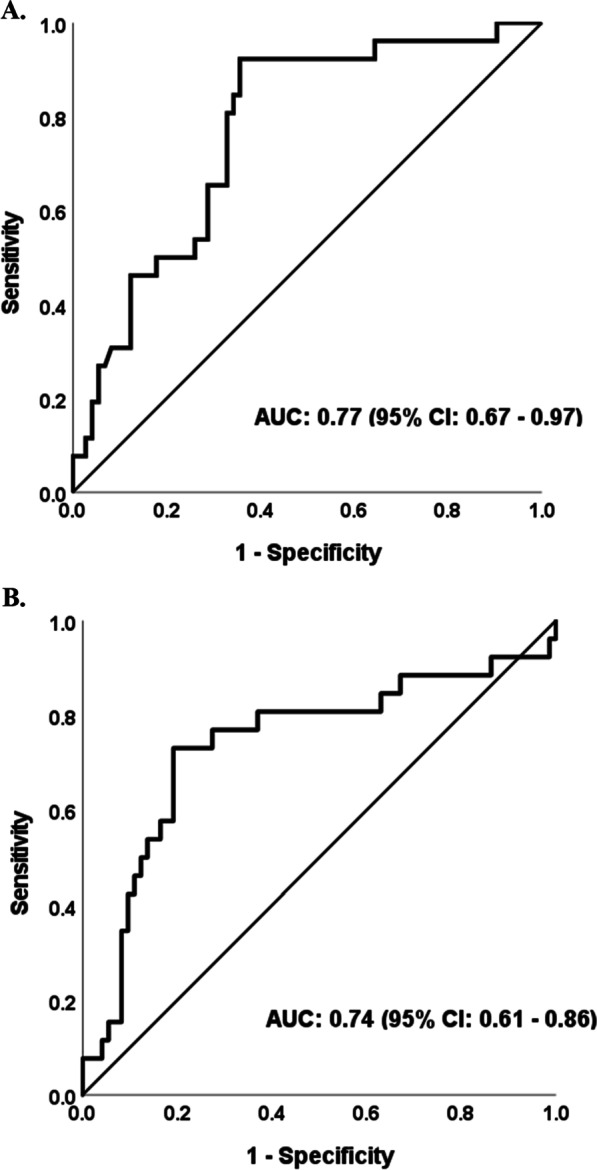
Receiver operating characteristic curves for MMP-3 prediction of 90-day mortality in ARDS. Receiver operating characteristics of **A** MMP-3 concentration on day 3 and **B** change in MMP-3 concentration from baseline to day 3

For the primary outcome, an AUROC curve analysis of day 3 MMP-3 concentration had an AUROC of 0.77 (95% confidence interval (CI): 0.67–0.87) for the prediction of 90-day mortality with an optimal cutoff value of 18.4 ng/mL (YI: 0.58) yielding a sensitivity of 92% and specificity of 63% (Fig. 1). Day 3 MMP-3 concentrations were significantly elevated in non-survivors at 90 days compared to survivors (26.4 ng/mL vs. 13.4 ng/mL, p < 0.001). Patients with elevated MMP-3 had fewer VFDs (11 days vs. 18 days, p = 0.003) and fewer ICU-free days (11.5 vs. 22, p = 0.01). Table 2 summarizes these results.

**Table 2:** Outcomes by MMP-3 concentration and change in MMP-3 from day 0 to 3

	Day 3 MMP-3 concentration		P-value	Day 0 to 3 MMP-3 difference		P-value
	High (≥ 18.4 ng/mL) (n = 50)	Low (< 18.4 ng/mL) (n = 50)		High (≥ 9.4 ng/mL) (n = 33)	Low (< 9.4 ng/mL) (n = 67)	
Outcome						
Mortality at 30 days	16 (32)	2 (4)	< 0.001	12 (36)	6 (9)	0.02
Mortality at 60 days	21 (42)	2 (4)	< 0.001	16 (48)	7 (10)	0.001
Mortality at 90 days	24 (48)	2 (4)	< 0.001	19 (58)	7 (10)	0.001
ICU free days	11 (0–21)	18 (11–23)	0.003	8 (0–17)	18 (10.5–22)	0.001
VFD	11.5 (0–22)	22 (14–24)	0.01	20 (10–23)	18.5 (0–22)	0.001
MMP-3 concentration						
Day 0	17.2 (11.7–24.3)	8.5 (4.6–12.1)	0.001	13.6 (9.3–21)	11.3 (5.5–17)	0.04
Day 3	27.9 (23.4–44.6)	11 (6.4–13.4)	0.001	34.4 (24.7–51.7)	12.9 (8.2–21.3)	0.001
Change day 0 to 3	+ 13.5 (+ 7.9 to + 23.3)	+ 0.7 (− 1.6 to + 4.2)	0.001	+ 17.5 (+ 13.5 to + 28)	+ 2.1 (− 1.5 to + 6.1)	0.001

All data are presented as n (%), mean ± SD, and median (interquartile range) unless otherwise noted

ALI: acute lung injury; ARDS: acute respiratory distress syndrome; APACHE III: Acute Physiology and Chronic Health Evaluation III; ICU: intensive care unit; MMP-3: matrix metalloproteinase-3; Vfd: ventilator free days

**Fig. 2:**
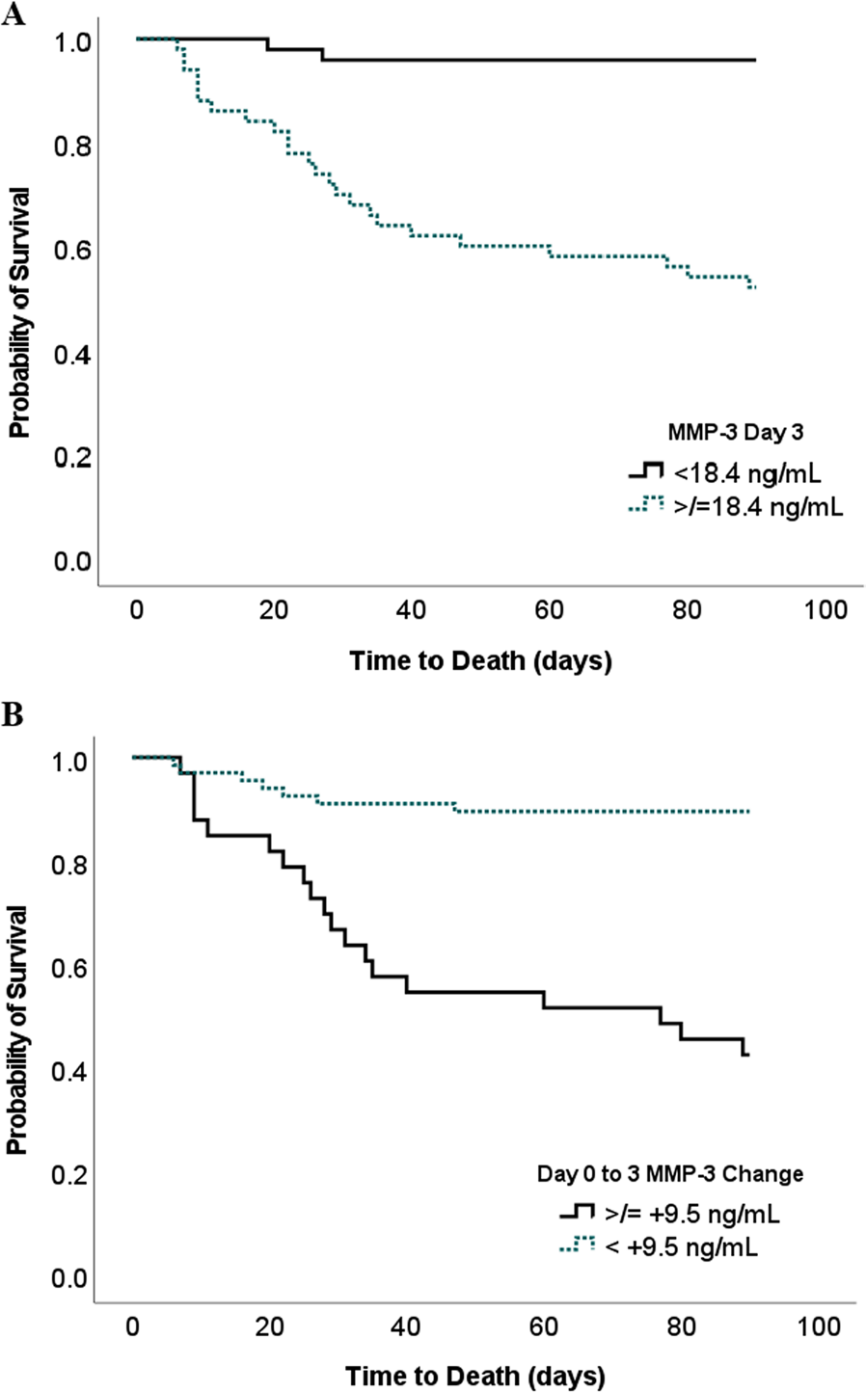
Kaplan–Meier survival curves stratified by MMP-3 concentration and change in MMP-3. **A** Day 3 MMP-3 concentration plotted as a survival curve separated into two groups by using the 18.4 ng/mL cutoff for day 3 MMP-3.** B** Day 0 to 3 MMP-3 concentration change plotted as a survival curve separated into two groups by using the 9.5 ng/mL cutoff for day 0 to 3 MMP-3 change. The probability of survival at 90 days was 95.9% vs 52% (P < 0.001) for low. vs. high MMP-3 concentration and 90% vs 42% (P < 0.001) for a change in MMP-3 from day 0 to 3 <  + 9.5 ng/mL and ≥  + 9.5 ng/mL, respectively

Among patients with day 3 MMP-3 ≥ 18.4 ng/mL, 48% died at 90 days, while among those with MMP-3 values below 18.4 ng/mL, 4% died at 90 days (p < 0.001). The probability of survival at 90 days was 96% vs. 52% (p < 0.001) for patients with < 18.4 ng/mL. vs. ≥ 18.4 ng/mL day 3 MMP-3 concentrations and 90% vs. 42% for a change in MMP-3 from day 0 to 3 <  + 9.5 ng/mL and ≥  + 9.5 ng/mL, respectively. Figure 2 displays Kaplan–Meier survival plots. In multivariate linear regression controlling for APACHE III score, MMP-3 concentration on day 3 was associated with 90-day mortality (OR: 1.024 [95% CI 1.004–1.045]), indicating each increase in 1 ng/mL predicted a 2.4% mortality increase (Table 3).

**Table 3:** Association of MMP-3 and APACHE III with mortality by multivariate regression

	Univariate		Multivariate	
	OR (95% CI)	P-value	OR (95% CI)	P-value
Day 0 MMP-3 concentration	1.022 (0.983–1.052)	0.28	1.001 (0.98–1.02)	0.89
Day 3 MMP-3 concentration	1.030 (1.008–1.052)	0.007	1.024 (1.004–1.045)	0.026
Day 0 to 3 MMP-3 change	1.005 (0.991–1.019)	0.46	0.999 (0.981–1.017)	0.89
APACHE III	1.032 (1.013–1.051)	< 0.001	1.028 (1.008–1.048)	0.005

MMP-3 variables were all individually tested in logistic regression with APACHE III score as covariates. Model variables that were iteratively removed stepwise included vasopressor use within the 24 h before randomization, PaO_2_/FiO_2_ at randomization, sex, and body mass index

APACHE III: Acute Physiology and Chronic Health Evaluation III; MMP-3: matrix metalloproteinase-3

The change in baseline MMP-3 was also explored as a predictor of mortality. The change from MMP-3 from day 0 to 3 was elevated among those with mortality at 90 days (+ 14.5 ng/mL vs. + 3.7 ng/mL, p < 0.001). Day 0 to 3 MMP-3 change was predictive of mortality with an AUROC of 0.74 and an optimal cutoff value of + 9.5 ng/mL (YI: 0.54), providing 73% sensitivity and 81% specificity (Fig. 1). Univariate and multivariate regression did not detect a significant association between Day 0 to 3 MMP-3 change and 90-day mortality.

### MMP-3 as a marker of ARDS

Additionally, MMP-3 concentrations in 20 healthy control patient samples were analyzed as a negative control. AUROC analysis of healthy controls and ALTA subjects, day 3 MMP-3 showed a high predictive value for ARDS with an AUROC of 0.86 (95% CI 0.76–0.93) and an optimal cutoff value of 9.9 ng/mL (YI, 0.75) with 80% sensitivity and 95% specificity (Additional file 1: Fig. S1). The 20 healthy samples showed significantly lower MMP-3 concentration than day 0 MMP-3 (6.5 ng/mL vs. 12.1 ng/mL, p < 0.001). Additional file 3: Table S2 reports total concentrations as medians and means of ALTA samples and healthy controls.

## Discussion

In this first analysis of the biomarker of MMP-3 from a randomized controlled trial of ARDS, MMP-3 performed well as a prognostic biomarker in ARDS, appropriately classifying patients with a higher risk of mortality and morbidity as measured by AUROC. Plasma MMP-3 levels as both static and dynamic measures showed marked elevations in non-survivors versus survivors, and multivariate regression identified a positive association with MMP-3 day concentrations and 90-day mortality when controlling for severity of illness. Moreover, MMP-3 was elevated in ARDS vs. non-ARDS patients.

The prognostic performance of MMP-3 was similar to a previous latent class analysis (LCA) of two randomized controlled ARDS trials (AUROCs ~ 0.75) [25]. This similar performance of a *single* biomarker is compared to a validated panel of clinical and biomarker variables, which may pose a superior strategy for diagnosing and prognosticating ARDS both as a single variable and an addition to current models [25]. Notably, the complex and heterogenous pathophysiology characterized by numerous acute phase reactants makes identification of a single, highly efficacious marker that is sufficiently powerful (i.e., AUROC > 0.9) for diagnosis and prognosis unlikely [25, 26]. However, these results support the hypothesis that lung-specific biomarkers may improve predictive power and/or model parsimony. Indeed, such a lung-specific biomarker may serve as an early (if imperfect) marker for disease that can reduce time to diagnosis (and thus time to intervention, particularly those that show maximal benefit in the early stages of ARDS), especially if used in the context of existing models and phenotyping efforts.

Beyond diagnosis, phenotyping using a biomarker, transcriptomic, and clinical data has shown promise to improve prognostication efforts [26]. Specifically, a dichotomous classification system has emerged with hyperinflammatory and hypoinflammatory phenotypes. The hyperinflammatory ARDS phenotype is characterized by shock, sepsis, and worse outcomes, while the hypoinflammatory phenotype occurs commonly in trauma-associated ARDS with better outcomes owed to features of rapidly improving ARDS [27, 28]. Across five separate phenotyping studies, hyperinflammatory phenotypes were suggested to have a 90-day mortality rate of 38%-51%, while hypoinflammatory phenotypes showed a rate of 17–23% [29]. Compared to the current study, the mortality rate in the high MMP-3 arms was similar to the hyperinflammatory phenotype, whereas the low MMP-3 arm had only 4% mortality despite comprising 50% of the cohort. Patients with more pronounced changes in MMP-3 from baseline to day 3 also had an increased risk of 90-day mortality, potentially implying a function of the intensity of MMP-3 elevations on disease progression; however, this study is unable to assess if MMP-3 is marker or a mediator for lung damage.

Differences in treatment response based on phenotype may explain the litany of negative results characteristic of ARDS treatment studies. Famous et al. showed the benefit of the fluid restriction intervention in ARDS occurs only in the hyperinflammatory phenotype and potentially worse outcomes in the non-inflammatory phenotype [30]. Using the same two phenotypes, Calfee et al. found simvastatin was associated with improved survival in the hyperinflammatory phenotype [31]. Recently, after the ROSE trial challenged routine use of neuromuscular blockade in ARDS, a reanalysis of the ROSE trial data suggested the inflammatory ARDS phenotype may benefit from neuromuscular blockade [32]. The present study did not aim to evaluate or establish phenotypes and phenotypic responses to treatments as no differences were observed with albuterol treatment in the overall cohort and this study used a small sample size of the larger study. An evaluation of albuterol’s effects on MMP-3 was beyond the scope of this investigation. Albuterol has repeatedly shown minimal clinical effects on mechanically ventilated patients, and thus even with larger sample sizes, no benefit is likely to exist [23, 33, 34]. However, biomarkers like MMP-3 related to ARDS pathophysiology and disease progression may aid in evaluating responses to treatment and support clinical trial enrichment by identifying patients most likely to benefit from a therapy, especially when combined with additionally clinical variables and biomarkers [26].

Multiple mechanisms linking MMP-3 to lung injury have been identified. Multiple MMPs contribute to ARDS pathogenesis, and MMP-3 has been shown as the primary driver of inflammatory MMP profiles [35]. MMP-3 is also mechanistically associated with ARDS outcomes as the impetus for MMP-3 production in lung endothelial cells is hyperinflammatory states [20, 36], the phenotype associated with worse outcomes. The mechanisms of MMP-3 mediated injury includes induction of epithelial-mesenchymal transition in lung epithelial cells [37], TGF-β1 activation [38], and junctional protein degradation, which are components of ARDS progression [14]. Additionally, MMP-3 has been associated with the progression of COVID-19 severity, and inflammatory cytokines are known to increase dramatically with COVID-19 [21, 39].

While many biomarkers have been associated with ARDS, few ARDS biomarkers have been suggested as therapeutic targets, including the receptor for advanced glycation end products (RAGE) [40, 41], club cell secretory protein (CC16) [8], and MMP-3 [14, 35]. Distinct from other ARDS biomarkers, MMP-3 has been linked preliminarily to a mainstay intervention in ARDS as neuromuscular blockade with *ci*satracurium reduced lipopolysaccharide induction of MMP-3 in human endothelial cells [42]. Interestingly, dexamethasone has an inhibitory effect on MMP-3 and other MMP activity [43–45]. Investigations into treatment effects of dexamethasone based on MMP-3 levels are an intriguing avenue for study given dexamethasone’s mortality reducing effects in ARDS [5, 46].

Finally, most investigations have evaluated variables at a single time point, assuming that early presentation is a reasonable predictor of overall outcome and treatment response. Yet, critical illness is known to be a highly dynamic state [47, 48]. However, Bhavani et al. recently published novel sepsis phenotyping that captured the dynamic nature of critical illness [49]. These models studied changes in vital signs over time (termed group-based trajectory changes) and identified a differential treatment response favoring balanced crystalloids compared to normal saline in one of the four subphenotype groups most characterized by persistent hypotension [49]. In the present study, dynamic assessments also yielded insights, as change over time of MMP-3 may provide an assessment of disease progression as increases in MMP-3 from baseline to day 3 were ubiquitous among non-survivors at 90 days. The period from baseline to day 3 may represent the early exudative phase of ARDS during which diffuse alveolar damage occurs, and MMP-3 pathogenesis is most present [1, 14], and importantly, the time crucial to initiate mortality-reducing ARDS interventions (e.g., lung protective ventilation, corticosteroids) [50, 51].

Strengths of this study included the use of clinical ARDS samples from a large randomized controlled trial, the evaluation of MMP-3 at multiple time points, and the novelty of using MMP-3 to predict ARDS mortality. The utility of MMP-3, particularly in a biomarker panel, may best be seen in its ability to guide clinically complex decisions: e.g., if patients with high MMP-3 who were treated with *cis*atracurium and/or dexamethasone had better outcomes than similar patients with MMP-3 without *cis*atracurium and/or dexamethasone. This scenario is hypothetical at present but shows the potential of such a biomarker to inform therapy. Despite these strengths, several limitations warrant discussion. First, the sample size and timing of collection may have limited the power to detect a more robust AUROC, especially for dynamic variables. No samples were available from the biorepository on days 1 and 2 (this study used only day 0 and 3); therefore, change in MMP-3 in the acute exudative phase of ARDS on days 1 through 2 were not captured. Second, the population had a small portion of trauma patients, with most patients having ARDS from infectious causes, which may bias the study towards the hyperinflammatory phenotype and prevent assessment of how MMP-3 responds in non-infectious ARDS (or the hypoinflammatory phenotype). Although MMP-3 showed strong differentiation capacity between ARDS and non-ARDS, the non-ARDS samples came from healthy patient samples, limiting the specificity for ARDS. Future diagnostic studies would be strengthened by evaluating critically ill patients with non-ARDS diagnoses. Finally, the ALTA trial was conducted from 2007 to 2008, and patient samples were frozen for approximately 15 years. Storage time is known to influence protein quality and yield, but the extent is not well described; however, plasma samples stored for 30 years can have ~ 35% of their protein concentration variation accounted for by storage time [52]. These samples likely have undergone some protein degradation, and concentrations would be expected to be higher than observed in this study.

## Conclusion

In conclusion, plasma MMP-3 levels demonstrated a prognostic relationship to ARDS mortality. Additionally, MMP-3 elevations from baseline may represent a phenotype of patients with elevated mortality risk. MMP-3 warrants further evaluation as a lung-specific biomarker for predicting treatment benefits among interventions known to improve mortality in ARDS. Future studies should include MMP-3 as a component in phenotyping and predictive methods.

## Supplementary Information

**Additional file 1: Figure S1.** Receiver operating characteristic curve for Day 3 MMP-3 prediction of ARDS. Data utilized to construct the curve were from 20 healthy non-diseased plasma samples and 100 ARDS samples from the ALTA trial on day 3 of enrollment.

**Additional file 2: Table S1**. Demographics and outcomes among ALTA trial treatment groups

**Additional file 3: Table S2.** MMP-3 Concentrations Medians and Means

## Data Availability

The datasets used and/or analysed during the current study are available from the corresponding author on reasonable request.
