# Supplementary material for: Plasma matrix metalloproteinase-3 predicts mortality in acute respiratory distress syndrome: a biomarker analysis of a randomized controlled trial
Source: Respir Res. 2023 Jun 22;24:166. doi: 10.1186/s12931-023-02476-5 (PMC10286483; doi:10.1186/s12931-023-02476-5)

**Figure S1.** Receiver operating characteristic curve for Day 3 MMP-3 prediction of ARDS. Data utilized to construct the curve were from 20 healthy non-diseased plasma samples and 100 ARDS samples from the ALTA trial on day 3 of enrollment.


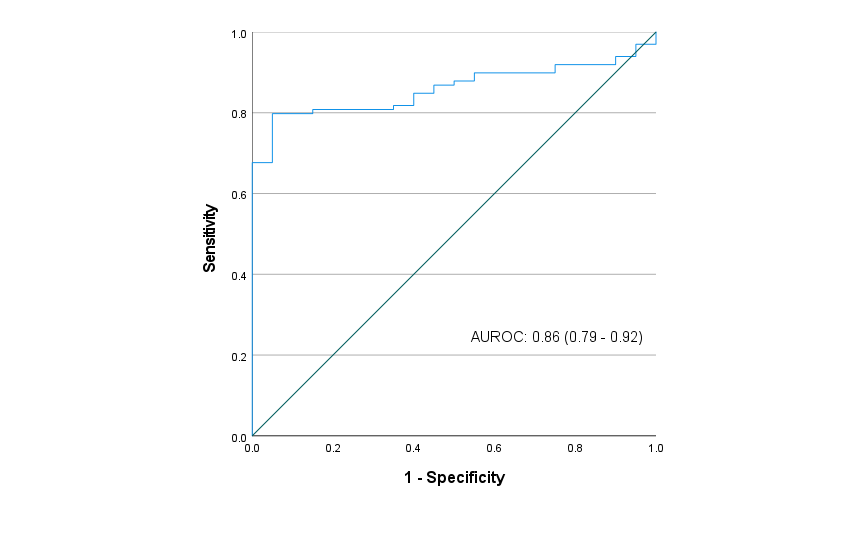

Supplement: Supplementary file 1 — Additional file 1: Figure S1. Receiver operating characteristic curve for Day 3 MMP-3 prediction of ARDS. Data utilized to construct the curve were from 20 healthy non-diseased plasma samples and 100 ARDS samples from the ALTA trial on day 3 of enrollment. [file 12931_2023_2476_MOESM1_ESM.docx]
