# Supplementary material for: Plasma matrix metalloproteinase-3 predicts mortality in acute respiratory distress syndrome: a biomarker analysis of a randomized controlled trial
Source: Respir Res. 2023 Jun 22;24:166. doi: 10.1186/s12931-023-02476-5 (PMC10286483; doi:10.1186/s12931-023-02476-5)
Supplement: Supplementary file 2 — Additional file 2: Table S1. Demographics and outcomes among ALTA trial treatment groups [file 12931_2023_2476_MOESM2_ESM.docx]

**Table S1**. Demographics and outcomes among ALTA trial treatment groups

|  | Trial treatment group | |  |
| --- | --- | --- | --- |
|  | Placebo  (n=50) | Albuterol  (n=50) | P-value |
| **Characteristic** |  | | |
| Age (years) | 52 ± 15 | 49 ± 16 | 0.33 |
| Male | 26 (52) | 28 (56) | 0.84 |
| Body mass index | 28±6 | 28 ±7 | 0.69 |
| APACHE, mean (SD) | 90±30 | 94±27 | 0.50 |
| Vasoactive use within 24 hours before randomization | 24 (48) | 28 (56) | 0.55 |
| Time from ALI to randomization (hours), median | 15 (10-31) | 23 (12-35) | 0.39 |
| PaO_2_/FiO_2_ | 140±60 | 144±60 | 0.77 |
| ARDS causes, n (%) |  | | |
| Pneumonia | 22 (44) | 16 (32) | 0.16 |
| Sepsis | 12 (24) | 16 (32) | 0.37 |
| Aspiration | 4 (8) | 12 (24) | 0.03 |
| Trauma | 8 (16) | 2 (4) | 0.047 |
| Multiple transfusions | 2 (4) | 0 | 0.55 |
| Other | 2 (4) | 4 (8) | 0.4 |
| **Outcome** |  | | |
| Mortality at 30 days | 7 (14) | 11 (22) | 0.44 |
| Mortality at 60 days | 10 (20) | 13 (26) | 0.64 |
| Mortality at 90 days | 11 (22) | 15 (30) | 0.50 |
| ICU free days | 17 (10-22) | 15 (0-21) | 0.07 |
| VFD | 20 (10-23) | 18.5 (0-22) | 0.07 |
| MMP-3 concentration |  | | |
| Day 0 | 11.9 (8.1-18.5) | 12.5 (6.4-17.9) | 0.93 |
| Day 3 | 17.2 (11-26.3) | 21.2 (10.4-28.8) | 0.89 |
| Change day 0 to 3 | +4.2 (+0.66 - +14.5) | +6.3 (-0.9 - +13.5) | 0.79 |
| All data are presented as n (%), mean ± SD, and median (interquartile range) unless otherwise noted.  ALI = acute lung injury, ARDS = acute respiratory distress syndrome, APACHE III = Acute Physiology and Chronic Health Evaluation III, ICU = intensive care unit, MMP-3 = matrix metalloproteinase-3, Vfd = ventilator free days | | | |
