# Supplementary material for: Plasma matrix metalloproteinase-3 predicts mortality in acute respiratory distress syndrome: a biomarker analysis of a randomized controlled trial
Source: Respir Res. 2023 Jun 22;24:166. doi: 10.1186/s12931-023-02476-5 (PMC10286483; doi:10.1186/s12931-023-02476-5)
Supplement: Supplementary file 3 — Additional file 3: Table S2. MMP-3 Concentrations Medians and Means [file 12931_2023_2476_MOESM3_ESM.docx]

**Table S2 MMP-3 Concentrations Medians and Means**

| MMP-3 Concentrations Medians and Means | | | | |
| --- | --- | --- | --- | --- |
| MMP-3 concentration | Total MMP-3 ng/mL  (n=100)  (n=20) healthy | High Day 3 MMP3 (≥18.4 ng/mL)  (n=50) | Low Day 3 MMP-3 (<18.4 ng/mL)  (n=50) | P-value* |
| Day 0 ALTA samples | 12.08 (7.33 – 18.49)  17.79 ± 36.05 | 17.2 (11.7– 24.3)  26.2 ± 49.4 | 8.5 (4.6– 12.1)  8.8 ± 4.7 | 0.001 |
| Day 3 ALTA samples | 19.20 (10.98 – 28.15)  26.10 ± 36.05 | 27.9 (23.4– 44.6)  41.7 ± 34.6 | 11 (6.4 – 13.4)  10.2 ± 4.5 | 0.001 |
| Change day 0 to 3 ALTA samples | 6.07 (0.074 – 13.55)  8.52 ± 38.19 | +13.5 (+7.9- +23.3)  +15.53 ± 52.92 | +0.7 (-1.6 – +4.2)  1.37 ± 4.08 | 0.001 |
| Healthy controls | 6.53 (4.93 – 9.50)  7.12 ± 2.46 |  |  |  |

*Comparisons are made between high and low MMP-3 day 3 groups

Data represented as medians (interquartile range) and mean ± standard deviation
